# The miR-21-5p, miR-30c-5p, and miR-182-5p as Biomarkers in Clear Cell Renal Cell Carcinoma: A Southeastern Romanian Cohort Study

**DOI:** 10.3390/genes16060650

**Published:** 2025-05-28

**Authors:** Ionuț Burlacu, Mariana Așchie, Georgeta Camelia Cozaru, Mariana Deacu, Gabriela Miruna Vizireanu, Adrian Nelutu Mitroi, Anca Florentina Mitroi, Costel Stelian Brînzan

**Affiliations:** 1 Pathology Department, Sf. Apostol Andrei Clinical Emergency County Hospital, 145 Tomis Blvd, 900591 Constanta, Romania; burlacuionut82@yahoo.com (I.B.); aschiemariana@yahoo.com (M.A.); drcozaru@yahoo.com (G.C.C.); deacu_mariana@yahoo.com (M.D.); branzancostel@yahoo.com (C.S.B.); 2 CEDMOG Center, Ovidius University, 145 Tomis Blvd., 900527 Constanta, Romania; gabriela.miruna@yahooo.com (G.M.V.); mitroi_74@yahoo.com (A.N.M.); 3 Faculty of Medicine, Ovidius University, 145 Tomis Blvd., 900527 Constanta, Romania; 4 CFR Hospital Constanta, Boulevard 1 Mai 3-5, 900123 Constanta, Romania

**Keywords:** clear cell renal cell carcinoma, miR-21-5p, miR-30c-5p, miR-182-5p, exosomes, qRT-PCR, non-invasive diagnosis

## Abstract

Background: This study aimed to evaluate the expression and clinical relevance of three mature miRNAs (miR-21-5p, miR-30c-5p, and miR-182-5p) in patients with clear cell renal cell carcinoma (ccRCC) from southeast Romania, and to explore their potential as non-invasive diagnostic and prognostic biomarkers. Methods: miRNA expression levels were measured using TaqMan^®^ MGB and qRT-PCR in paired tumor and adjacent non-cancerous tissues, as well as in serum-derived exosomes, from 26 ccRCC patients. Statistical analysis included the Wilcoxon test for group comparisons and non-parametric tests for correlations with clinicopathological features. Receiver operating characteristic (ROC) curves were used to assess diagnostic performance, and miRNA panels were constructed for improved accuracy. Results: Significant dysregulation of the investigated miRNAs was observed. miR-21-5p was markedly overexpressed in both tumor tissues (3.46-fold, *p* < 0.001) and serum exosomes (3.26-fold, *p* < 0.001). miR-182-5p showed modest overexpression in tissues (0.56-fold, *p* < 0.001) and serum (0.85-fold, *p* < 0.001), whereas miR-30c-5p was significantly downregulated in both tissues (2.48-fold decrease, *p* < 0.001) and serum exosomes (2.29-fold decrease, *p* = 0.0003). Elevated miR-182-5p expression correlated with tumor localization in the right kidney (*p* = 0.02) and lymph node involvement (*p* = 0.04). Similarly, higher miR-21-5p levels in serum exosomes were associated with right-sided tumors (*p* = 0.01). ROC analysis revealed distinct expression profiles for all three miRNAs between ccRCC and normal tissue, both in tissue and exosomal samples (all *p* < 0.05). Combined biomarker panels yielded high diagnostic performance (AUC = 0.94 for tissue, AUC = 0.93 for exosomes). Conclusions: This study underscores the potential of miR-21-5p, miR-30c-5p, and miR-182-5p as non-invasive biomarkers for ccRCC diagnosis and prognosis. The use of serum exosomal miRNA panels offers a promising alternative to tissue-based diagnostics in Romanian ccRCC patients.

## 1. Introduction

Renal cell carcinoma (RCC) stands as the prevailing type of kidney cancer, constituting approximately 3 to 5% of all newly diagnosed cancer cases. According to GLOBOCAN estimates, Romania recorded 2.750 new cases of RCC and 1.066 deaths in 2020, ranking 11th and 15th, respectively [1]. RCC comprises a set of different histologies, within which the clear cell subtype is the most prevalent (ccRCC), accounting for 75–80%, followed by the papillary (pRCC) and chromophobe subtypes (chRCC) [2].

The data on the incidence and mortality of RCC in different geographical regions emphasize the need to implement regional screening programs and develop accurate biomarkers [3]. The current gold standard for the diagnosis of kidney cancer is pathological biopsy, which is an invasive method [4]. The classical triad of abdominal pain, visible hematuria, and palpable abdominal masses is now rarely observed in patients with RCC [5]. Instead, many RCC cases are incidentally discovered during routine imaging procedures such as abdominal magnetic resonance imaging (MRI), computed tomography (CT), or ultrasonography [6]. However, it is important to note that these methods are generally unsuitable for systematic screening purposes. The landscape of RCC screening presents significant challenges in modern healthcare, with various diagnostic approaches showing both promise and limitations. Current screening protocols rely heavily on imaging technologies, though no standardized screening guidelines exist for the general population [7].

A promising category of emerging biomarkers comprises small non-coding microRNAs (miRNAs), owing to their widespread presence in bodily fluids, tissue-specific expression patterns, and robust stability. miRNAs regulate gene expression at the post-transcriptional level by silencing diverse messenger ARN (mRNA) targets and governing a hub of biological processes [8]. While numerous studies underscore the prognostic and diagnostic relevance of miRNAs present in both tissue and blood samples in the context of RCC, published findings often display considerable variability and occasionally conflicting data regarding specific miRNAs [9].

Extracellular vesicles (EVs), especially exosomes, are emerging as valuable biomarkers released by nearly all cell types. These nanometer-sized spherical vesicles comprise various molecular elements, such as proteins, lipids, and nucleic acids, with non-coding RNAs (ncRNAs) being particularly notable. This group includes miRNAs, long non-coding RNAs (lncRNAs), and circular RNAs (circRNAs) [10,11]. Importantly, exosomal biomarkers present clear advantages over other circulating biomarkers due to their lipid bilayer, which safeguards them against proteolytic and nucleolytic degradation [12]. Additionally, exosomes are thought to accurately represent the pathophysiological condition of their source cells because of their distinct biogenesis [13]. Numerous studies have confirmed the considerable potential of exosomal miRNAs for diagnosing and predicting various cancers, including RCC [14,15]. Despite this, only a few studies have directly analyzed the diagnostic effectiveness of freely circulating versus exosomal miRNAs [16].

This study aimed to investigate the clinical utility of three mature miRNAs (hsa-miR-21-5p, hsa-miR-30c-5p, and hsa-miR-182-5p) as potential biomarkers in RCC. This analysis was conducted using 26 tissue and serum exosomal samples collected from patients diagnosed with ccRCC from the southeastern region of Romania. The selected miRNAs were chosen based on their documented clinical relevance in carcinogenesis, as evidenced by prior studies [17,18,19].

## 2. Materials and Methods

### 2.1. Patients, and Collection of Tissue and Serum Samples

The study examined 26 pairs of fresh cancerous renal tissue and adjacent non-cancerous renal tissue (NRC) sourced from patients who underwent radical nephrectomy at the Urology Department of the Emergency County Clinical Hospital in Constanța, Romania, between June 2019 and November 2021. A pathologist selected representative areas from the tumors after surgical removal and sliced them for histological examination and miRNA molecular analysis. Additionally, preoperative peripheral blood samples were acquired from the same patients using BD Vacutainer^®^ SST™ II Advance Tubes. None of the patients had undergone chemotherapy or radiotherapy before the surgery. Both tissue and serum samples were immediately preserved in RNAlater solution (Invitrogen, Thermo Fisher Scientific, Waltham, MA, USA) to stabilize RNA species, then stored at −80 °C until extraction. Histopathological evaluation of the ccRCC samples applied the Fuhrman grading system, and clinical staging followed the American Joint Committee on Cancer (AJCC) anatomical staging guidelines. A control group of 14 healthy individuals was also included in the study. Ethical approval was provided by the Local Ethics Commission for Clinical and Research Developmental Studies, and all participants gave written informed consent prior to sample collection.

### 2.2. Extraction of miRNAs from Tissues

Small RNA molecules were isolated from the samples, starting with approximately 25–30 mg of tissue. Initially, tissue was homogenized in a 700 mL lysis buffer using a miRNeasy kit (Qiagen, Hilden, Germany) for 90 s. After homogenization, 140 mL of chloroform was added, and the samples were incubated at room temperature for 5 min. Afterward, centrifugation was performed at 12,000 rpm for 15 min at 4 °C to separate the upper aqueous phase containing the RNA. This RNA was then precipitated by adding 1.5 volumes of 100% ethanol. The resulting precipitated samples were transferred into an RNeasy Mini column, followed by two successive centrifugation steps involving 2 wash buffers (RW1 and RPE). For the final elution step, the column was carefully placed into a new tube, and 30 mL of RNase-free water was added. Subsequently, the mixture was centrifuged at maximum speed for 1 min to collect the eluate.

### 2.3. Preparation of Blood Samples

To maintain high pre-analytical quality, blood samples were processed within one hour of collection, ensuring optimal serum preservation. After allowing 20 min for clot formation at room temperature (RT), the tubes underwent centrifugation for 10 min at 3000 rpm and 4 °C. This step involved separating the upper yellow serum phase from the remaining blood components and transferring it to a new Eppendorf tube, ensuring the intermediate leukocyte layer remained undisturbed. A visual inspection was conducted on all samples for signs of discoloration or contamination, and hemolysis of serum was measured using a NanoDrop One spectrophotometer. Any affected samples were excluded from subsequent analysis. To eliminate additional cellular nucleic acids adhering to cell debris, the serum samples underwent another centrifugation at 3000 rpm and 4 °C for 15 min. The resulting supernatant was aliquoted in nuclease-free cryotubes and stored at −80 °C for further processing.

### 2.4. Exosomal miRNA Extraction

Exosomes were enriched from serum using the miRCURY^®^ Exosome Serum/Plasma Kit (Qiagen, Hilden, Germany). Briefly, 1.4 mL of cleared serum was mixed with 560 μL of Precipitation Buffer A in a microcentrifuge tube. Following overnight incubation at 2–8 °C, the precipitate was centrifuged at 1500× *g* for 30 min at RT. The resulting supernatant was discarded, and the pellet was resuspended in 240 μL of resuspension buffer. Subsequently, 1 mL of QIAzol Lysis Reagent was added to the resuspended RNA pellets, vortexed, and left to stand for 5 min at RT. From this point, the extraction steps were consistent with those described for miRNA extraction from tissues.

**Figure 1:**
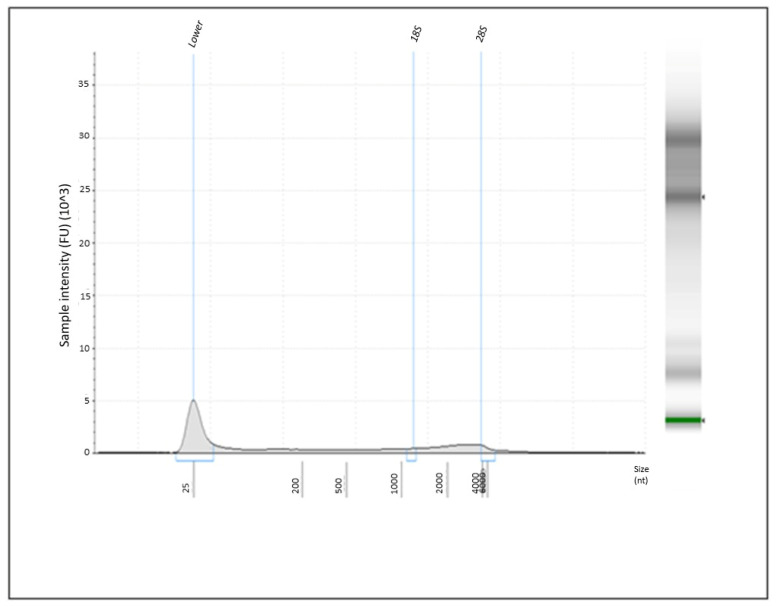
The image illustrates the identification of serum exosomal miRNA species isolated from patients with ccRCC and controls. Serum exosomes were purified using miRCURY Exosome Kits (Qiagen), and miRNAs were separated and analyzed using a 2200 TapeStation Bioanalyzer (Agilent Technologies GmbH, Germany) in conjunction with the RNA HS ScreenTape kit. Abundant miRNAs were identified, typically around 25 nucleotides in length, while 18S and 28S ribosomal RNAs were scarcely detected.

The quality and quantity of the RNA solutions were assessed using various methods. Purity was measured with a NanoDrop One Spectrophotometer (Thermo Fisher Scientific, Waltman, MA, USA), where an acceptable A260/A280 ratio ranges from 2 to 2.1, and the A260/A230 ratio should be above 2. Sample concentration was determined using a Qubit 3.0 Fluorometer (Thermo Fisher Scientific, Waltman, MA, USA) and the Qubit RNA HR Assay Kit. Exosomes contain a variety of RNA types, including messenger RNAs (mRNAs), transfer RNAs (tRNAs), ribosomal RNAs (rRNAs), and miRNAs. After isolation, exosomal miRNAs were separated and analyzed with a 2200 TapeStation Bioanalyzer (Agilent Technologies GmbH, Waldbronn, Germany) alongside the RNA HS ScreenTape kit. Notably abundant miRNAs, generally around 25 nucleotides, were identified, while 18S and 28S ribosomal RNAs were seldom detected (Figure 1). These results demonstrate that our isolation technique effectively enriched miRNAs and confirmed that the exosomes extracted from serum samples were predominantly rich in miRNAs, making them ideal for further clinical sample analysis.

### 2.5. Reverse Transcription of miRNA to Complementary cDNA and the qPCR Reaction

Selected mature human miRNAs were converted into complementary DNA (cDNA) using the TaqMan miRNA Reverse Transcription Kit, supplied by Applied Biosystems. In each reaction, a specific RNA-specific stem–loop primer was employed to initiate reverse transcription, as outlined in Table 1. The RNA concentration was maintained between 1 and 10 ng for every 15 μL of reaction (consisting of 7 μL of a master mix, 3 μL of primer, and 5 μL of the RNA sample). These samples were then incubated in a TProfessional Basic Gradient Thermocycler (Biometra GmbH, Gottingen, Germany): 16 °C for 30 min, 42 °C for 30 min, and 85 °C for 5 min, followed by cooling to 4 °C. To synthesize the complementary DNA strand, a TaqMan MGB Assay with a specific sequence was used. In a 20 μL reaction mix, 10 μL of TaqMan 2× Universal PCR Master Mix was combined with 1.33 μL of the product obtained from the RT reaction, along with 7.67 μL of RNase-free H_2_O and 1 μL of TaqMan Small RNA assay (20X).

Quantitative real-time polymerase chain reaction (qPCR) analysis was conducted in duplicate for each sample, employing the ABI 7500 Fast qPCR instrument for 40 cycles. Each cycle included a denaturation step at 95 °C for 3 s, followed by an annealing step at 60 °C for 30 s, culminating in primer extension and probe cleavage, with fluorescence detection at the end of each cycle. Negative controls without templates were used in all runs. The average cycle threshold (Ct) values from duplicates of each miRNA and endogenous control (RNU44 and miR-16-5p) were calculated using automatic baseline and threshold settings with the 7500 Fast Real-Time PCR software version 2.3. The selection of RNU44 for tissue samples and miR-16-5p for serum exosomes was based on manufacturer recommendations (Applied Biosystems TaqMan^®^ MicroRNA Assays) and prior literature demonstrating stable expression under our experimental conditions [20]. The relative expression levels of miR-21-5p, miR-30-5p, and miR-182-5p were determined by the Livak equation: 2-(ΔCt), where ΔCt = Ct_miR target_ − Ct_RUN44_ for tissue and ΔCt = Ct_miR target_ − Ct_miR16_ for serum exosomes.

### 2.6. Statistical Analysis

Statistical analysis and graphical representations were made using MedCalc^®^ Software, version 14.8.1 (MedCalc Software Ltd., Ostend, Belgium).

The normality of the Gaussian distribution was assessed using the D’Agostino–Pearson test, and in instances where the resulting *p*-value was below 0.05, non-parametric tests were employed. Quantitative variables were described with measures such as range, mean, standard deviation, and median, while qualitative variables were presented as frequencies and percentages.

To determine the statistical differences in miRNA species between groups, Wilcoxon tests were applied, with statistical significance defined as a *p*-value less than 0.05. Receiver operating characteristic (ROC) analysis was utilized to assess the diagnostic performance of the selected miRNAs as potential biomarkers for distinguishing RCC cases from normal cases. The diagnostic accuracy was evaluated in terms of the area under the curve (AUC), sensitivity (Sn), and specificity (Sp).

## 3. Results

### 3.1. The Clinical and Demographic Variables of Participants

The study involved two groups: a cohort of twenty-six ccRCC patients (eighteen men, eight women; mean age: 67.5 years, range: 42–85) from whom tissue and serum samples were collected, and a control group of fourteen healthy individuals (seven men, seven women; mean age: 70 years, range: 40–85) from whom serum samples were collected. No significant differences in age or gender distribution were observed between the ccRCC cohort and the control group (*p* > 0.05).

The mean size of the tumors was 5.96 cm (range: 2–12 cm), with 53.84% (14) located in the left kidney and 46.16% (12) in the right kidney. Regarding tumor depth, 46.15% (12) of tumors were classified as T1/T2, while 53.85% (14) were classified as T3/T4. In terms of nodal and metastasis status, 65.38% (seventeen) were categorized as N0/Nx, while 34.62% (nine) were classified as N1. Similarly, 61.54% (16) were in M0/Mx, and 38.46% (10) were in M1. For the Fuhrman pathological grade, tumors were equally distributed between the low-grade (G1/G2: 50%) and high-grade (G3/G4: 50%) categories.

### 3.2. The Expression Level of Selected miRNAs in Tissue and Serum Exosome Samples from ccRCC Patients

Our study investigated the expression levels of three mature miRNAs (miR-21-5p, miR-30c-5p, and miR-182-5p) in a cohort of 26 ccRCC tissues and serum exosomal samples, utilizing various normalization strategies for miRNA analysis. In the tissue analysis, miRNA expression in ccRCC tumors was compared to adjacent normal renal tissue from the same patients. For serum exosomal miRNA levels, normalization was carried out against a control group of age- and sex-matched healthy individuals who had no cancer history or significant comorbidities.

**Figure 2:**
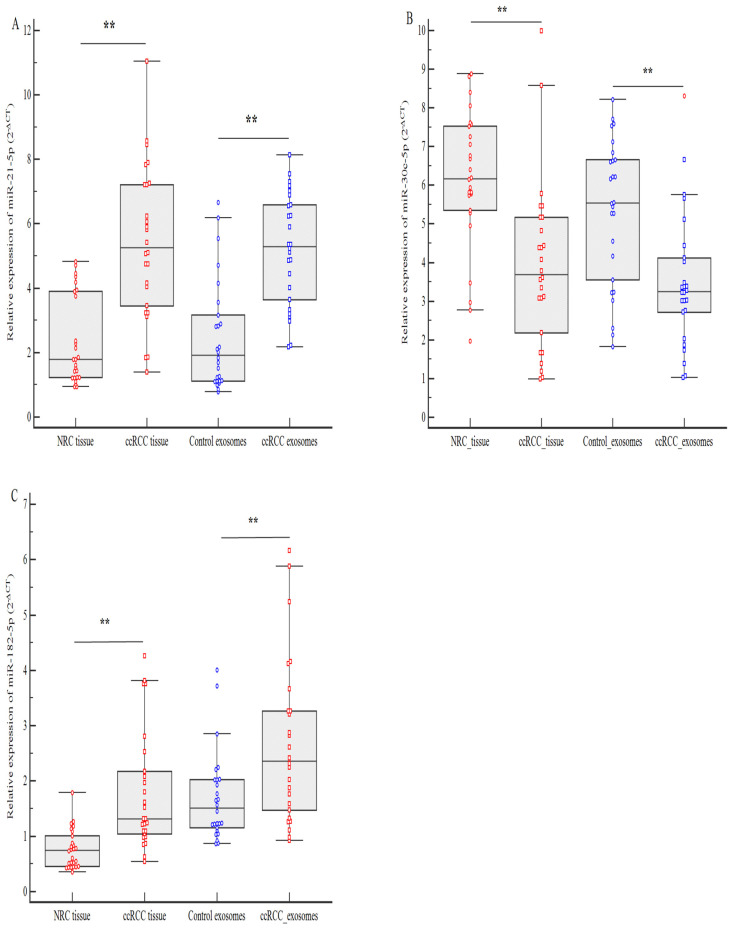
Expression levels of miRNAs (2−ΔΔCt) in ccRCC patients box plot: (**A**) Relative expression of miR-21-5p in tissue samples and serum exosomes. (**B**) Relative expression of miR-30c-5p in tissue samples and serum exosomes. (**C**) Relative expression of miR-182-5p in tissue samples and serum exosomes. Expression levels were analyzed by qRT-PCR in triplicate and normalized to RNU44 (tissue samples) and miR-16-5p (serum exosomes), which served as endogenous controls. *p* values were calculated using the paired Wilcoxon signed-rank test (** <0.001).

The findings indicated a notable dysregulation of miRNAs in ccRCC patients. Both miR-21-5p and miR-182-5p exhibited upregulation, while miR-30c-5p showed marked downregulation. As illustrated in Figure 2, miR-21-5p expression was significantly increased in ccRCC tissue compared to normal renal tissue (NRC), with a 3.46-fold increase (*p* < 0.0001). Likewise, serum exosomal levels of miR-21-5p were substantially higher in ccRCC patients compared to controls, showing a 3.28-fold increase (*p* < 0.0001). Additionally, miR-182-5p displayed a slight increase in expression in ccRCC tissue versus NRC (0.56-fold, *p* < 0.001), with elevated levels found in serum exosomes (0.85-fold, *p* = 0.003) compared to healthy controls. In contrast, pairwise comparisons revealed that miR-30-5p was significantly downregulated across all sample types: in tissue (2.48-fold reduction, *p* < 0.001) and serum exosomes (2.29-fold decline, *p* = 0.0003).

### 3.3. Correlation of Selected miRNA Expression in Tissues and Serum Exosomes with Clinicopathological Characteristics

To explore the clinical relevance of the selected miRNAs, their expression levels were compared with clinicopathological characteristics (Table 2). Certain features were grouped for analysis, including tumor size (<5 cm vs. ≥5 cm), Fuhrman grade (G1/G2 vs. G3/G4), pT stage (T1/T2 vs. T3/T4), pN stage (N0/Nx vs. N1), and pM stage (M0/Mx vs. M1). No statistically significant differences in miRNA expression were observed concerning gender, age, tumor size, tumor depth, metastasis, Fuhrman grade, and perineural/perivascular invasion.

However, higher expression of miR-182-5p was associated with tumor location in the right kidney versus the left kidney, both in tissue and serum exosomes (*p* = 0.02 for both). Similarly, elevated levels of miR-21-5p in serum exosomes were linked to right-sided tumors (*p* = 0.01). Additionally, high miR-182-5p expression showed a significant association with lymph node involvement (*p* = 0.04).

### 3.4. ROC Analysis

**Figure 3:**
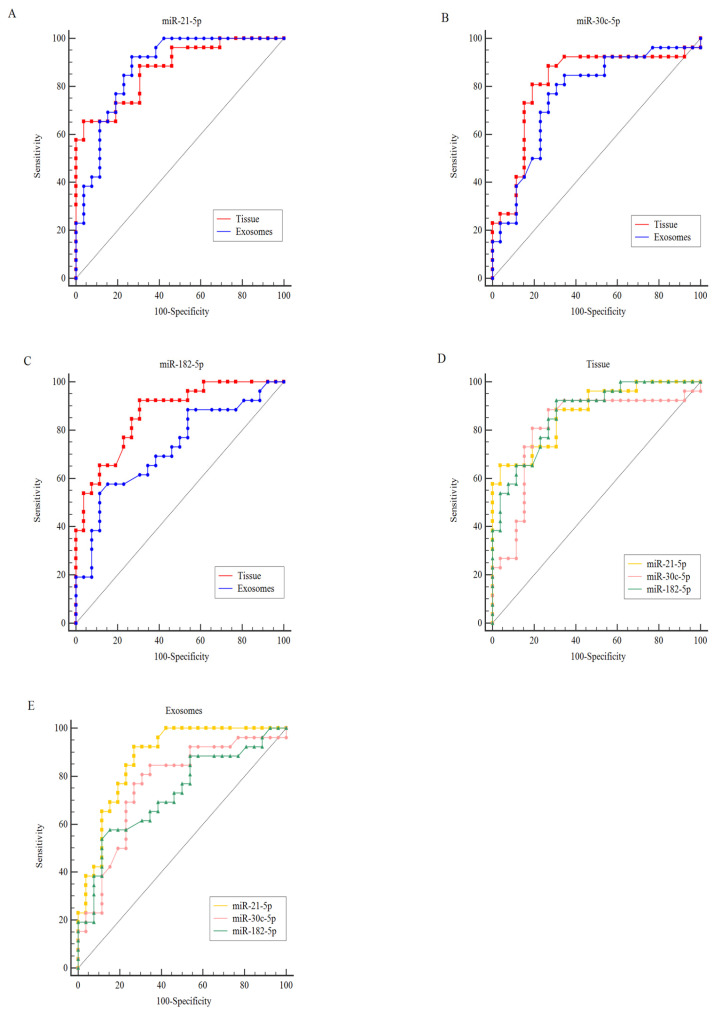
ROC curves were analyzed to evaluate the expression levels of miR-21-5p, miR-30c-5p, and miR-182-5p as potential biomarkers for ccRCC detection. The AUC for upregulated miR-21-5p (**A**) expression was 0.87 in tissue (Sn: 65.38%, Sp: 96.15%; *p* < 0.001) and 0.87 in exosomes (Sn: 92.31%, Sp: 73.08%; *p* < 0.001). For miR-182-5p (**B**), the AUC was 0.86 in tissue (Sn: 92.31%, Sp: 69.23%; *p* < 0.001) and 0.72 in exosomes (Sn: 57.69%, Sp: 84.62%; *p* < 0.001). For downregulated miR-30c-5p (**C**), the AUC was 0.81 in tissue (Sn: 80.77%, Sp: 80.77%; *p* < 0.001) and 0.76 in exosomes (Sn: 76.92%, Sp: 73.08%; *p* < 0.001). Additionally, the combined analysis of the three miRNAs (miR-21-5p, miR-30c-5p, and miR-182-5p) yielded an AUC of 0.94 in tissue (Sn: 96.15%, Sp: 92.31%; *p* < 0.001) (**D**) and 0.93 in the serum exosomal compartment (Sn: 80.77%, Sp: 100%; *p* < 0.001) (**E**).

The ability of miR-21-5p, miR-30c-5p, and miR-182-5p to serve as prognostic biomarkers was assessed by ROC and AUC (Figure 3 and Table 3).

Comparison between ccRCC and NRC groups revealed distinct expression profiles for all three selected miRNAs in both tissue samples and serum exosomes (all < 0.05). miR-21-5p exhibited the highest discriminatory power, with identical AUC values of 0.87 in tissues and serum exosomes. In tissues, miR-21-5p demonstrated a sensitivity of 65.38% and specificity of 96.15%, while in serum exosomes, these values shifted to 92.31% sensitivity and 73.08% specificity.

Similarly, miR-30c-5p showed robust diagnostic performance, achieving an AUC of 0.81 in tissues and 0.76 in serum exosomes. In tissues, sensitivity and specificity were both 80.77%, whereas in exosomes, sensitivity decreased slightly to 76.92% and specificity to 73.08%. For miR-182-5p, tissue analysis yielded a higher AUC (0.86) compared to serum exosomes (0.72). Tissue-based detection showed superior sensitivity (92.31%) but lower specificity (69.23%), while exosomal analysis prioritized specificity (84.62%) over sensitivity (57.69%).

To enhance diagnostic accuracy, panels combining miR-21-5p, miR-30c-5p, and miR-182-5p were constructed for both tissue and exosomal analyses (Table 4). The exosomal miRNA panel achieved an AUC of 0.93, closely matching the tissue panel’s performance (AUC = 0.94), underscoring the potential of combined biomarkers for reliable discrimination.

## 4. Discussion

The RCC has become an increasingly significant concern in recent years, with survival rates largely influenced by the clinical stage at diagnosis [21]. Therefore, early detection of RCC is critical for improving patient outcomes.

Importantly, the potential use of miRNAs as non-invasive biomarkers for RCC is increasingly recognized due to their role in post-transcriptional gene regulation and their presence in diverse biological samples. Compared to mRNAs, miRNAs exhibit superior stability (even under harsh conditions such as repeated freeze–thaw cycles), resistance to degradation by natural ribonucleases, ease of accessibility, and high abundance in bodily fluids, including blood and urine [22]. While tissue-derived miRNAs reflect localized expression patterns within tumors, circulating exosomal miRNAs provide a minimally invasive approach and may better capture tumor heterogeneity through systemic distribution.

In this study, we evaluated the feasibility of three mature miRNAs, miR-21-5p, miR-30c-5p, and miR-182-5p, as potential biomarkers for ccRCC by analyzing renal tissue and serum exosomes from 26 RCC patients. All three miRNAs showed significant dysregulation in both tissue and serum samples, reinforcing their potential utility as non-invasive tools for early diagnosis.

miR-21-5p is a key oncomiR, often overexpressed in various solid and hematological malignancies, where it is crucial in carcinogenesis [17]. Our previous study demonstrated that the oncogenic role of miR-21-5p is significantly linked to several cancer types, including colorectal cancer, indicating its potential as a tissue biomarker for this disease [23]. In RCC, research has revealed that miR-21-5p is notably overexpressed in the ccRCC and pRCC subtypes when compared to healthy kidney tissue and benign renal tumors [17]. In agreement with our earlier work, we detected a substantial increase in miR-21-5p levels in ccRCC tissues (3.46-fold) and serum exosomes (3.26-fold). The alignment of miR-21-5p levels in both tissue and circulating exosomes supports the idea that tumor-derived exosomes may act as a reservoir for miR-21-5p, facilitating its release into the blood. This reinforces its potential as a non-invasive diagnostic marker. The strong AUC values (0.87 in both matrices) further confirm miR-21-5p’s ability to distinguish between conditions. However, there were notable differences in sensitivity and specificity between tissues and exosomes. The higher sensitivity in exosomes may indicate that they capture tumor heterogeneity through systemic distribution more effectively than localized tissue samples, while the greater specificity in tissues likely arises from direct tumor sampling. Despite the diagnostic potential of miR-21-5p, its correlations with clinicopathological parameters such as tumor size, TNM stage, or Fuhrman grade were limited. This is consistent with findings by Gaudelot et al., who noted a similar disconnect between miR-21-5p expression and disease progression in ccRCC [24]. Interestingly, the significant association between elevated exosomal miR-21-5p levels and tumor laterality, specifically right-sided localization (*p* = 0.01), suggests potential anatomical or molecular distinctions in ccRCC pathogenesis or tumor microenvironment interactions. These findings underscore the need to investigate the underlying mechanisms.

miR-30c-5p, a key member of the miRNA-30 family, is notably underexpressed in various cancers and is recognized as a multifunctional regulator of cell proliferation, differentiation, metabolism, and apoptosis [25]. In general, miR-30c-5p, considered a tumor suppressor miRNA, was significantly downregulated in all analyzed samples from our study, both in tumor tissues (2.48 reduction) and in serum exosomes of ccRCC patients (2.29 decline). This suggests a potential role in inhibiting cell migration, invasion, and proliferation. Our findings align with previous research that identified miR-30c-5p downregulation in several cancer types, including bladder cancer and invasive micropapillary carcinoma [26]. The prognostic value of miR-30c-5p was confirmed by ROC analysis, with AUC values ranging from 0.76 in exosomes to 0.81 in tissues, indicating that the tissue biomarkers are more effective in distinguishing between classes. In tissue, sensitivity and specificity were equal, suggesting a well-balanced model with strong discriminatory power. In exosomes, sensitivity and specificity were relatively close to each other, indicating a moderately strong classifier. No statistically significant associations were found between miR-30c-5p levels and clinicopathological parameters. These results are consistent with the findings of Onyshchenko et al., who reported miR-30c-5p downregulation in tumor tissues [27].

miR-182-5p, part of the miR-183 family, is a well-studied miRNA involved in various biological functions, and its dysregulation is associated with tumorigenesis and other diseases [28]. While our studies found miR-182-5p to be overexpressed in ccRCC, it was to a lesser extent than miR-21-5p. miR-182-5p’s elevated levels in serum exosomes showed significant correlations with tumor location in the right kidney (*p* = 0.02) and lymph node involvement (*p* = 0.04). These findings suggest a potential role for miR-182-5p in tumor progression and lymphatic spread, which might enhance our understanding of the metastatic mechanisms in ccRCC. The lower diagnostic performance observed in exosomes may indicate technical difficulties in detecting low-abundance miRNAs or biological variations in how exosomes are packaged. Sensitivity and specificity assessments revealed a stark contrast between tissues and exosomes. The tissue-based approach indicated high sensitivity, making it useful for disease detection, while the exosome-based method showed greater specificity. Although miR-182-5p is often considered oncogenic in various cancers [22], our results differ from those indicating downregulated miR-182-5p in ccRCC. This inconsistency may arise from differences in sample types (e.g., tissue versus circulating exosomes), methodological discrepancies, or tumor heterogeneity. More research is required to clarify these findings and better understand miR-182-5p’s context-dependent roles in ccRCC pathogenesis [29].

The integration of miR-21, miR-30c-5p, and miR-182-5p into biomarker panels significantly improved diagnostic accuracy, achieving AUCs of 0.93 (exosomes) and 0.94 (tissue), comprising higher values compared to individual markers. This combined miRNA panel also demonstrated excellent sensitivity and specificity, highlighting the synergistic value of multi-miRNA approaches in mitigating the limitations of individual markers. Exosomal miRNAs, in particular, offer non-invasive advantages, with their performance nearly matching tissue-based analysis. This underscores the potential of using a panel of biomarkers, rather than a single miRNA, to enhance diagnostic accuracy. These findings support their inclusion in future rapid diagnostic assays for ccRCC. As a next step, we aim to validate our results in a multicenter study involving larger and ethnically diverse patient cohorts and to assess the specificity of these miRNAs against other genitourinary malignancies (e.g., bladder and prostate cancer) to determine their RCC-specific diagnostic value.

This study has several limitations. First, due to limited regional patient availability, the relatively small sample size may lack sufficient statistical power to thoroughly assess associations between miRNA expression levels and specific pathological features. A formal power analysis was not conducted, as this was an exploratory pilot study; however, the statistically significant results observed support the robustness of the initial findings. To address these limitations, larger-scale, prospective studies with diverse cohorts are warranted to validate these preliminary findings and strengthen the generalizability of the results.

## 5. Conclusions

In conclusion, this study underscores the diagnostic potential of miR-21-5p, miR-30c-5p, and miR-182-5p as non-invasive biomarkers for ccRCC. Significant dysregulation of all three miRNAs was observed in both tumor tissues and serum exosomes, with miR-21-5p showing a robust overexpression, miR-30c-5p acting as a tumor suppressor through marked downregulation, and miR-182-5p exhibiting slight overexpression in comparison with miR-21-5p.

The diagnostic performance of individual miRNAs highlights their utility in distinguishing ccRCC from non-cancerous conditions. Furthermore, combining these miRNAs into a panel significantly enhanced diagnostic accuracy, achieving AUCs of 0.93 (exosomes) and 0.94 (tissue), emphasizing the advantage of multi-marker approaches. The near-equivalent performance of exosomal miRNAs to tissue-based analysis further supports their viability as non-invasive tools, offering practical advantages for early detection while capturing systemic tumor heterogeneity.

## Figures and Tables

**Table 1:** The mature miRNA sequence.

miRNA	Mature miRNA Sequence
hsa-miR-30c-5p	UGUAAACAUCCUACACUCUCAGC
hsa-miR-21-5p	UAGCUUAUCAGACUGAUGUUGA
hsa-miR-182-5p	UUUGGCAAUGGUAGAACUCACACU
hsa-miR-16-5p	CCAAUAUUACUGUGCUGCUUUA
RNU44	UAUUGCACUUGUCCCGGCCUG

**Table 2:** Analysis of miR-21-5p, miR-30c-5p, and miR-182-5p with the clinicopathological features of CRC patients.

Clinicopathological Variables		No. of Patients (%)	miR-21-5p		miR-30c-5p		miR-182-5p	
			Tissue P; χ^2^	Exosomes P; χ^2^	Tissue P; χ^2^	Exosomes P; χ^2^	Tissue P; χ^2^	Exosomes P; χ^2^
Gender	Male	18 (69.23)	0.73; 0.11	0.73; 0.11	0.06; 3.51	0.67; 0.17	0.53; 0.38	0.61; 0.26
	Female	8 (30.77)						
Age	≥65	16 (61.54)	0.10; 2.59	0.08; 2.95	0.72; 0.12	0.97; 0.001	0.48; 0.48	1.00; 0.00
	<65	10 (38.46)						
Tumor location	Left	14 (53.85)	0.05; 3.78	0.01; 6.07	0.14; 2.12	0.21, 1.55	0.02; 5.08	0.02; 5.32
	Right	12 (46.15)						
pT	T1/T2	11 (42.31)	0.47; 0.50	0.73; 0.11	0.67; 0.17	0.70, 0.14	0.17; 1.82	0.41; 0.67
	T3/T4	15 (57.69)						
pN	N0/Nx	17 (65.38)	0.37; 0.78	0.28; 1.14	0.77; 0.08	0.32, 0.97	0.04; 4.02	0.23; 1.39
	N1	9 (34.62)						
pM	M0/Mx	13 (50)	0.45; 0.56	0.97; 0.001	0.94; 0.005	0.79, 0.07	0.85; 0.03	0.89; 0.01
	M1	13 (50)						
Fuhrman Grade	G1/G2	13 (50)	0.63; 0.22	0.73; 0.11	0.78; 0.07	0.52, 0.40	0.38; 0.74	0.61; 0.24
	G3/G4	13 (50)						
IPN	IPN0/IPNx	24 (92.31)	0.52; 0.41	0.32; 0.96	0.51; 0.42	0.49, 0.46	0.76; 0.08	0.37; 0.78
	IPN1	2 (7.69)						
ILV	ILV0/ILVx	21 (80.77)	0.44; 0.58	0.86; 0.02	0.66; 0.19	0.75, 0.09	0.81; 0.05	0.44; 0.58
	ILV1	5 (90.23)						

**Table 3:** Receiver operating characteristics curve analysis of miR-21-5p, miR-30c-5p, and miR-182-5p.

miRNA	AUROC (CI; *p* Value)	Sensitivity	Specificity	Cut-Off
miR-21-Tissue	0.87 (0.75–0.94; *p* < 0.001)	65.38%	96.15%	0.61
miR-21-Exosomes	0.87 (0.75–0.95; *p* < 0.001)	92.31%	73.08%	0.65
miR-30c-5p-Tissue	0.81 (0.67–0.90; *p* < 0.001)	80.77%	80.77%	0.61
miR-30c-5p-Exosomes	0.76 (0.62–0.86; *p* < 0.001)	76.92%	73.08%	0.50
miR-182-5p-Tissue	0.86 (0.74–0.94; *p* < 0.001)	92.31%	69.23%	0.61
miR-182-5p-Exosomes	0.72 (0.58–0.84; *p* = 0.006)	57.69%	84.62%	0.42

**Table 4:** AUCs, sensitivity, and specificity for a combination of three miRNAs (miR-21-5p, miR-30c-5p, and miR-182-5p) in the tissue and serum exosomal compartment.

Combinations	AUCROC (CI; *p* Value)	Sensitivity	Specificity	Cut-Off
Tissue	0.94 (0.84–0.98; *p* < 0.0001)	96.15%	92.31%	0.88
Exosomes	0.93 (0.82–0.98; *p* < 0.0001)	80.77%	100%	0.80

## Data Availability

The data presented in this study are available on request from the authors.

## Abbreviations

The following abbreviations are used in this manuscript:

RCC	Renal cell carcinoma
ccRCC	Clear cell renal cell carcinoma
pRCC	Papillary renal cell carcinoma
chRCC	Chromophobe renal cell carcinoma
MRI	Magnetic resonance imaging
CT	Computed tomography
miRNA	MicroRNA
EV	Extracellular vesicles
ncRNA	Non-coding RNA
lncRNA	Long non-coding RNA
circRNA	Circular RNA
NCR	Adjacent non-cancerous renal tissue
AJCC	American Joint Committee on Cancer
mRNA	Messenger RNA
tRNA	Transfer RNA
rRNA	Ribosomal RNA
cDNA	Complementary DNA
RT	Reverse transcription
qPCR	Quantitative real-time polymerase chain reaction
CT	Cycle threshold
ROC	Receiver operating characteristic
AUC	Area under the curve
Sn	Sensitivity
Sp	Specificity
